# 6″-Debromohamacanthin A, a Bis (Indole) Alkaloid, Inhibits Angiogenesis by Targeting the VEGFR2-Mediated PI3K/AKT/mTOR Signaling Pathways 

**DOI:** 10.3390/md11041087

**Published:** 2013-04-02

**Authors:** Gi Dae Kim, Oug Jae Cheong, Song Yi Bae, Jongheon Shin, Sang Kook Lee

**Affiliations:** 1 Natural Products Research Institute, College of Pharmacy, Seoul National University, San 56-1 Sillim-dong, Gwanak-gu, Seoul 151-742, Korea; E-Mails: gidaekim@snu.ac.kr (G.D.K.); baesy722@hanmail.net (S.Y.B.); shinj@snu.ac.kr (J.S.); 2 Department of Chemistry, McGill University, Montreal, Quebec, H3A 2K6, Canada; E-Mail: Ougjaecheong@gmail.com

**Keywords:** 6″-debromohamacanthin A, anti-angiogenesis, PI3K/AKT/mTOR, HUVEC, mouse embryonic stem cells

## Abstract

Hamacanthins, bis (indole) alkaloids, are found in a few marine sponges, including *Spongosorites* sp. Hamacanthins have been shown to possess cytotoxic, antibacterial and antifungal activities. However, the precise mechanism for the biological activities of hamacanthins has not yet been elucidated. In the present study, the anti-angiogenic effects of 6″-debromohamacanthin A (DBHA), an active component of isolated hamacanthins, were evaluated in cultured human umbilical vascular endothelial cells (HUVEC) and endothelial-like cells differentiated from mouse embryonic stem (mES) cells. DBHA significantly inhibited vascular endothelial growth factor (VEGF)-induced cell proliferation, migration and tube formation in the HUVEC. DBHA also suppressed the capillary-like structure formation and the expression of platelet endothelial cell adhesion molecule (PECAM), an endothelial biomarker, in mES cell-derived endothelial-like cells. To further understand the precise molecular mechanism of action, VEGF-mediated signaling pathways were analyzed in HUVEC cells and mES cell-derived endothelial-like cells. DBHA suppressed the VEGF-induced expression of MAPKs (p38, ERK and SAPK/JNK) and the PI3K/AKT/mTOR signaling pathway. In addition, DBHA inhibited microvessel sprouting in mES/EB-derived embryoid bodies. In an *ex vivo* model, DBHA also suppressed the microvessel sprouting of mouse aortic rings. The findings suggest for the first time that DBHA inhibits angiogenesis by targeting the vascular endothelial growth factor receptor 2 (VEGFR2)-mediated PI3K/AKT/mTOR signaling pathway in endothelial cells.

## 1. Introduction

Angiogenesis is the formation of new blood vessels from pre-existing vessels. Angiogenesis is a multistep process that includes the destabilization of an established vessel and endothelial cell proliferation, migration and tubulogenesis. In particular, tumor angiogenesis is the expansion of a network of blood vessels penetrating into cancerous growths to supply nutrients and oxygen and to remove metabolic waste from the tumors. Therefore, tumor growth and metastasis are highly dependent upon the angiogenic process [1]. In each step of this process, endothelial cells play a major role, such as endothelial cell proliferation, adhesion, migration, invasion and tube formation [2]. 

One of the most essential factors regulating angiogenesis is vascular endothelial growth factor (VEGF). VEGF acts on endothelial cells as both a chemotactic and a mitogenic factor via endothelial cell-specific receptors [3]. The functional role of VEGF is mediated by VEGF receptor (VEGFR)-1 (Flt-1, 180 kDa) and VEGFR-2 (KDR/Flk-1, 200 kDa), both of which are almost exclusively expressed in endothelial cells [4]. In particular, the biological activity of VEGF is mainly associated with VEGFR2-mediated signal transduction. The binding of VEGF to the VEGFRs and the subsequent ligand-induced dimerization of these receptors stimulate their intrinsic tyrosine kinase activity and trigger key angiogenic responses of endothelial cells, including proliferation, migration and differentiation via a number of signaling cascades [5]. In general, VEGF/VEFGR2-mediated signal transduction is accomplished by the activation of MAPKs including ERK, SAPK/JNK and p38 in endothelial cells. The phosphoinositide 3-kinase (PI3K)/AKT/mTOR signaling pathway is one of the most commonly activated signaling pathways in human cancer [6]. PI3K catalyzes the phosphorylation of the 3-hydroxyl position of PIP2 (phosphatidylinositol 4,5-diphosphate) to PIP3 (phosphatidylinositol 3,4,5-triphosphate). The deregulation of PI3K leads to elevated PIP3 levels and the downstream activation of AKT [7]. Mammalian target of rapamycin (mTOR), a serine/threonine protein kinase, also regulates cell growth, cell cycle progression and angiogenesis by the activation of the downstream translational regulators p70S6 kinase (p70S6K) and eukaryotic initiation factor (eIF) 4E binding protein 1 (4EBP1) [8]. Indeed, the activation of the MAPK/mTOR signaling pathway in endothelial cells increases their survival when cultured *in vitro* and in the tumor vasculature *in vivo* [9,10].

Platelet endothelial cell adhesion molecule (PECAM), a member of the immunoglobulin super-family, is expressed on both immature and mature endothelial cells and mediates cell–cell adhesion through either homotypic or heterophilic interactions [11]. PECAM also plays an important role in the early stages of vascular sprouting when new sprouts invade the extracellular matrix. Therefore, PECAM is considered a cell surface marker for endothelial cells and endothelial sprouting in vasculogenesis and angiogenesis.

Embryonic stem (ES) cells are pluripotent cell lines derived from the blastocyst-stage cells of early mammalian embryos. These unique cells are characterized by their extended undifferentiated proliferation in tissue culture and a prolonged potential to differentiate into a variety of cell types [12]. Therefore, the application of ES cells has been considered to be a potential tool for drug discovery. In addition, we recently established a 3-dimenional model system for the evaluation of angiogenesis that involves the differentiation of mouse ES cell-derived embryoid bodies into endothelial-like cells [13].

Indole-containing alkaloids have been frequently isolated from diverse marine invertebrates including bryozoans, coelenterates, sponges and tunicates. In particular, bis (indole) alkaloids are abundant in marine sponges. Hamacanthins, bis (indole) alkaloids, exhibit a variety of pharmacological activities, such as cytotoxicity, antitumor, antiviral, antimicrobial and anti-inflammatory activities [14,15,16,17,18,19,20,21,22,23]. In addition, we also reported that hamacanthins showed antibacterial, antifungal and cytotoxic activities [24,25]. However, studies of the functional effects of hamacanthins on angiogenesis and their mechanism of action in endothelial cells have not been undertaken.

**Figure 1:**
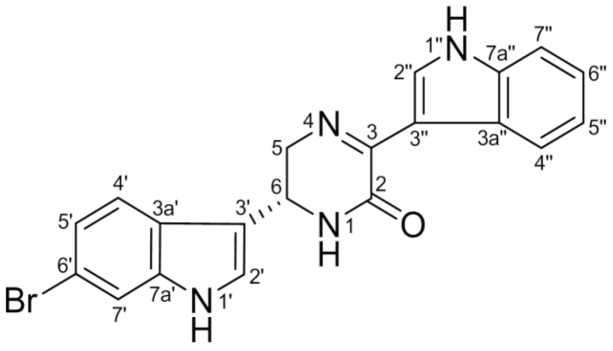
Chemical structure of 6″-debromohamacanthin A (DBHA) from the sponge *Spongosorites* sp.

In this study, we investigated the effects of 6″-debromohamacanthin A (DBHA, Figure 1), a representative hamacanthin from the marine sponge *Spongosorites* sp., on angiogenesis in endothelial cells and explored the underlying molecular mechanisms involved. We found that DBHA significantly inhibited angiogenesis in human umbilical vascular endothelial cells and mouse embryonic stem cell-derived endothelial-like cells as determined by the measurement of cell proliferation and migration, *in vitro* tube formation and *ex vivo* vascular sprouting. The anti-angiogenic activity was also associated with the suppression of the VEGFR2-mediated PI3K/AKT/mTOR signaling pathway.

## 2. Results and Discussion

### 2.1. DBHA Inhibits the Viability of VEGF-Induced HUVEC and mES-Derived Endothelial-Like Cells

**Figure 2:**
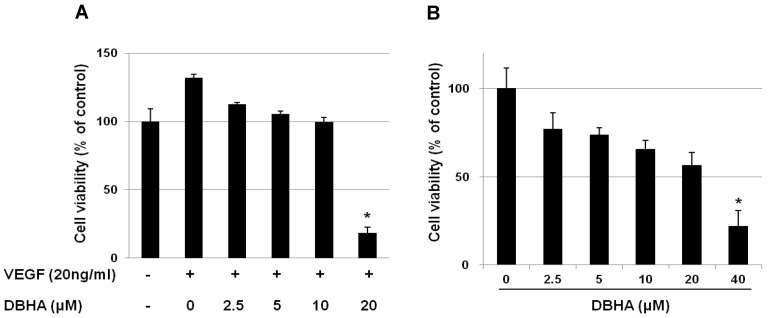
The inhibitory effects of DBHA on cell viability of human umbilical vascular endothelial cells (HUVEC) (**A**) and mES-derived endothelial-like cells (**B**). The HUVEC were cultured with DBHA (0–20 μM) in the presence of vascular endothelial growth factor (VEGF) (20 ng/mL) for 24 h. The differentiated mES-derived endothelial-like cells were cultured with DBHA (0–40 μM) for 24 h. Cell viability is expressed as the percentage compared to the vehicle-treated control and is expressed as the mean ± SD. * P < 0.05 compared to control.

Many angiogenesis inhibitors suppress endothelial cell proliferation *in vitro*. To determine the anti-angiogenic activity of DBHA, we first evaluated whether DBHA inhibits VEGF-induced proliferation of HUVEC and mES-derived endothelial-like cells. When treated with VEGF (20 ng/mL) for 24 h, the proliferation of the HUVEC increased by approximately 30% compared with that of the vehicle-treated control cells; however, DBHA significantly suppressed the VEGF-stimulated proliferation of the human endothelial cells in a concentration-dependent manner, with an IC_50_ value of 14.8 μM (Figure 2A). To determine the concentration of DBHA that does not induce cytotoxicity in the differentiated endothelial-like cells, the cells were initially treated with DBHA (0–40 μM) for 24 h, and the cell viability was then evaluated with the MTT assay. As shown in Figure 2B, DBHA did not exert a significant cell viability up to 10 μM, but over 20 μM DBHA exhibited a cytotoxic effect in mES cell-derived endothelial-like cells compared to control (IC_50_: 28.5 μM). Therefore, further analyses of the biological activities of DBHA were performed using less than a 10 μM concentration of DBHA in the differentiated endothelial-like cells.

### 2.2. DBHA Inhibits the VEGF-Induced Cell Migration and Tube Formation of HUVEC

**Figure 3:**
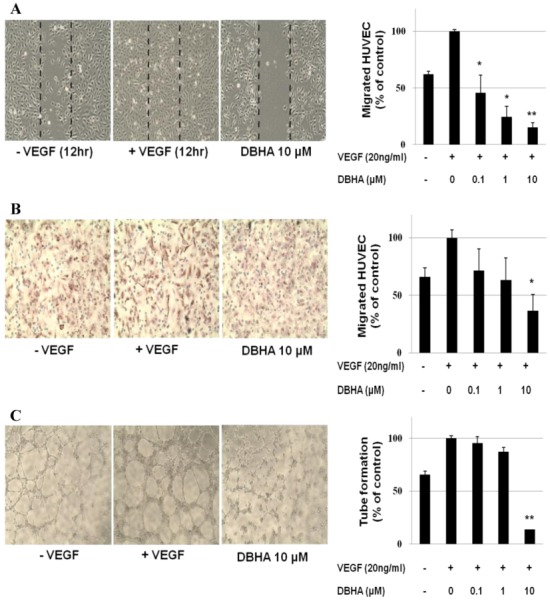
The effects of DBHA on the VEGF-induced cell migration and capillary structure formation of endothelial cells. (**A**) DBHA inhibited HUVEC migration. Cells were grown to confluency in six-well plates, wounded, and treated with the indicated concentrations of DBHA and VEGF (20 ng/mL). (**B**) DBHA inhibited endothelial cell migration in a transwell migration assay. HUVEC treated with various concentrations of DBHA were seeded in the upper chamber, and the bottom chamber was filled with endothelial growth medium EGM medium containing 20 ng/mL VEGF. The cells with an irregular shape in the images are cells that migrated into the lower chamber. (**C**) DBHA inhibited the VEGF-induced tube formation of HUVEC. Cells were placed in 96-well plates coated with Matrigel. After 4–8 h in the absence and presence of DBHA, the tubular structures were photographed. The migrated cells were quantified by manual counting. The results are reported as the mean ± SD. * *P* < 0.05, ** *P* < 0.01 *vs.* VEGF-stimulated cells.

Endothelial cell migration and tube formation are essential steps in angiogenesis. Based on this information, we determined the effects of DBHA on endothelial cell migration using both wound-healing and transwell cell migration assays *in vitro*. DBHA suppressed the migration of the VEGF-stimulated HUVEC in a concentration-dependent manner (Figure 3A). DBHA also inhibited the migration of the HUVEC in the Matrigel-coated transwell migration assay (Figure 3B). To further investigate the effect of DBHA on endothelial cells, we examined the VEGF-stimulated tube formation of the HUVEC in Matrigel. It is well known that endothelial cells are able to spontaneously form capillary-like network on Matrigel *in vitro*, an important feature in the process of angiogenesis [26]. As shown in Figure 3C, the HUVEC spontaneously formed a capillary-like tube structure after 4–8 h of incubation on Matrigel. However, the VEGF-stimulated HUVEC formed tube structures that were more prominent, with more stable and longer networks. DBHA, however, remarkably inhibited the VEGF-stimulated tubular network formation of the HUVEC, resulting in less elongated, broken and foreshortened tubes. These data suggested that DBHA inhibited the VEGF-induced capillary-like tube formation of endothelial cells. 

**Figure 4:**
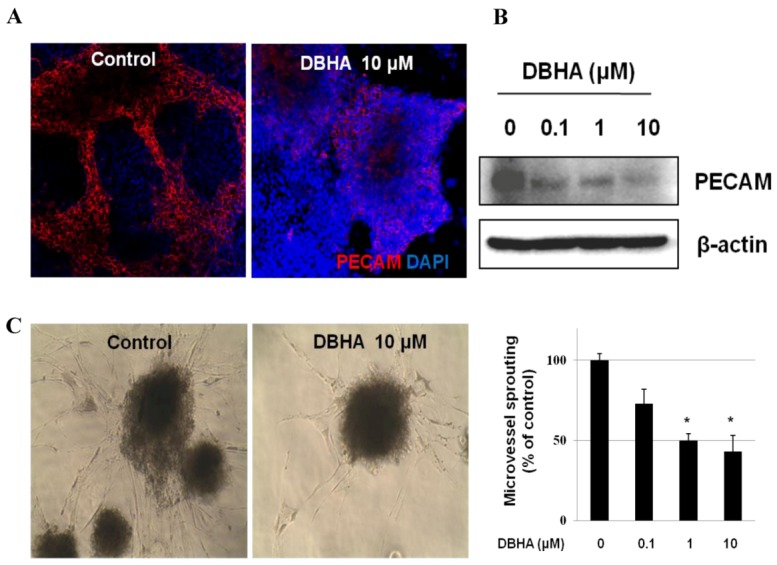
The inhibitory effects of DBHA on vessel formation. (**A**) mES cell-derived endothelial-like cells were exposed on day 10 to DBHA (0–10 μM) and incubated for 24 h. mES cell-derived endothelial-like cells stained with an antibody directed against the endothelial cell marker PECAM using immunofluorescence. Cell nuclei were stained with 4′,6-Diamidino-2-Phenylindole DAPI. (**B**) Platelet endothelial cell adhesion molecule (PECAM) proteins from different treatments were analyzed by a western blot assay. (**C**) The embryoid dodies (EBs) derived from mES cells were cultured in a suspension containing EGM-2 medium for 7 days. The EBs embedded in the collagen gel were then treated with DBHA for 4 days. The endothelial cell sprouting activity of EB-derived endothelial-like cells was altered with DBHA (0, 0.1, 1 or 10 μM) treatment. The morphology of the endothelial cell sprouting of the EB-derived endothelial-like cells was recorded by photography, and the sprouting was quantified by manual counting under a microscope. Statistical analysis was performed with Student’s *t*-test. * *P* < 0.05 *vs*. vehicle control. Magnification 40×.

### 2.3. DBHA Suppresses PECAM Expression and Capillary Sprouting of mES Cell-Derived Embryoid Bodies

To determine whether the DBHA-mediated inhibition of endothelial-like cell growth was associated with the suppression of endothelial cell biomarker expression, the embryoid body (EB)-derived endothelial cells (on day 10) were treated with DBHA (0–10 μM) for 24 h. After treatment, the expression of the endothelial cell biomarker PECAM was also assessed in a 2-dimensional (2-D) culture. In addition, the protein expression of the endothelial cell-specific biomarker PECAM was analyzed. As shown in Figure 4A, PECAM was easily detected by immunofluorescence in the mES/EB-derived endothelial cells, but DBHA (10 μM) effectively suppressed PECAM expression. In addition, as shown in Figure 4B, DBHA also suppressed the protein expression of PECAM in a concentration-dependent manner, with the expression of PECAM barely detectable after treatment with 10 μM DBHA. To further clarify whether DBHA affects the differentiation of endothelial progenitor cells into endothelial cells, the formation of blood vessel-like structures (microvessel sprouting) was investigated in a 3-dimensional (3D) culture of mES cell-derived embryoid bodies. As shown in Figure 4C, capillary sprouting manifested in the 3D culture of mES cell-derived embryoid bodies after 4 days of incubation (control), but the treatment of the cultures with DBHA remarkably inhibited the sprouting in a concentration-dependent manner. These data suggest that DBHA can effectively suppress the development of blood microvessel sprouting structures in EBs (3D culture). 

### 2.4. DBHA Inhibits Capillary Spouting in the Mouse Aortic Ring Assay

**Figure 5:**
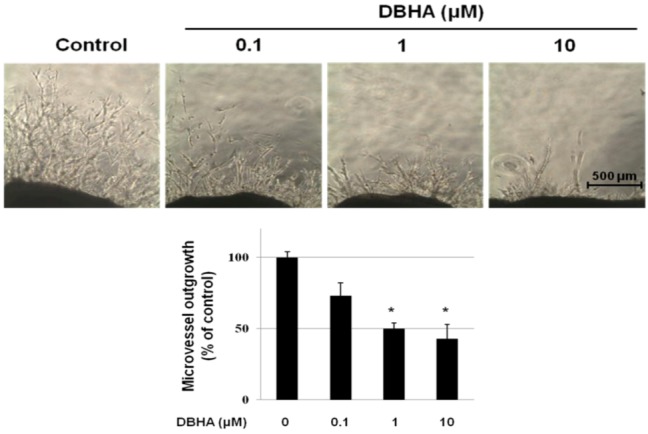
The effect of DBHA on microvessel outgrowth arising from mouse aortic rings. Aortic rings isolated from mice were embedded in Matrigel in 48-well plates and then fed medium containing various concentrations of DBHA for 7 days. Representative photographs of three independent experiments are shown. The bar equals 500 μm. The microvessel length was measured on day 7 of culture. The values are the means ± SD (*n* = 3), and * indicates *P* < 0.05 *vs.* control cells.

Further studies were designed to investigate the effect of DBHA in an *ex vivo* angiogenesis model using the mouse aortic ring assay. This assay system is widely used as an effective tool for evaluating the anti-angiogenic activity of test compounds in a complex system in which endothelial cells, fibroblasts, pericytes and smooth muscle cells interact [27]. Fibroblastic fusiform cells migrated from the ends of the aortic rings after 2–3 days, and the cells then spread in the Matrigel. Microvessels emerged from the ends of the aortic rings after 5–6 days and elongated. Compared with the vehicle-treated control group, the DBHA-treated aortic rings strongly suppressed the outgrowth of microvessel in a concentration-dependent manner, and a significant inhibition of microvessel sprouting was observed after treatment with 10 μM DBHA (Figure 5). 

**Figure 6:**
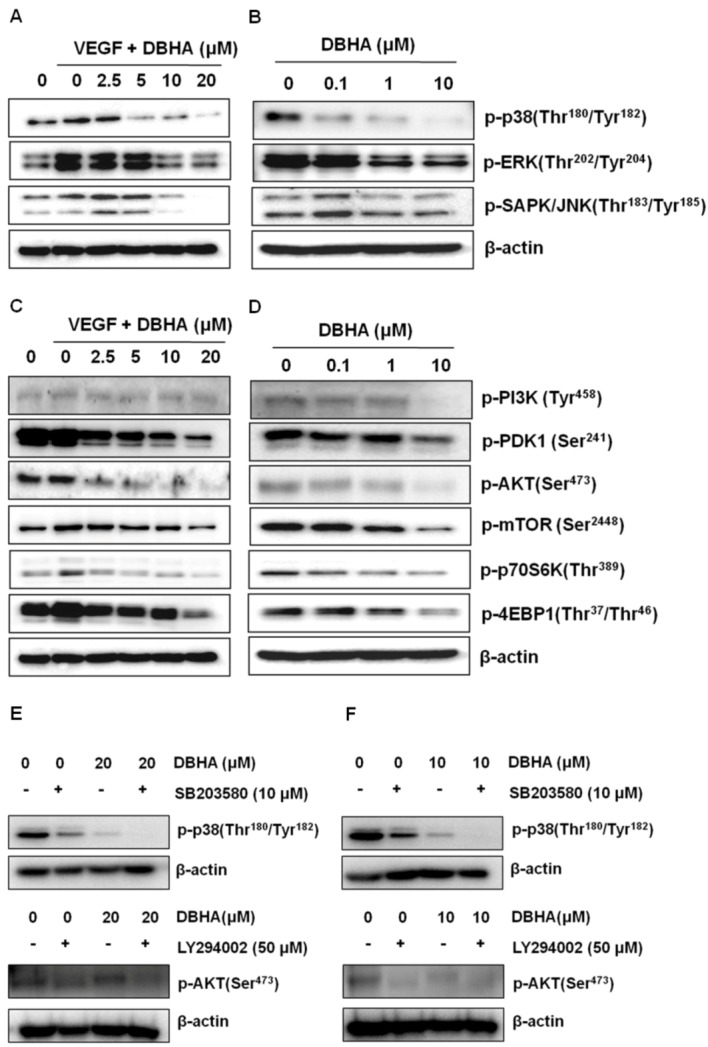
The molecular basis for the DBHA effects on angiogenesis. DBHA inhibited the phosphorylation of MAPK (**A**, **B**) and PI3K/AKT/mTOR (**C**, **D**) in HUVEC (**A**, **C**, **E**) and mES derived endothelial cells (**B**, **D**, **F**). After starvation in EGM without serum overnight, the HUVEC were washed twice with PBS and then incubated in the presence of VEGF containing (20 ng/mL) medium with DBHA for 24 h. mES derived endothelial-like cells were exposed on day 10 to DBHA (0–10 μM) and incubated for 24 h. (**E**, **F**) The effect of co-treatment of p38 inhibitor SB203580 and/or PI3K inhibitor LY294002 with DBHA on the expression of p-p38 and p-AKT. The proteins isolated from the cultures exposed to the different treatments were analyzed by a western blot assay. β-actin was used as an internal control.

### 2.5. DBHA Inhibits MAPKs and the PI3K/AKT/mTOR Signaling Pathway in VEGF-Stimulated HUVEC and mES-Derived Endothelial-Like Cells

To further understand the molecular basis of the DBHA-mediated anti-angiogenic activity, we next examined the modulation of VEGF-stimulated cellular signaling pathways in HUVEC and mES-derived endothelial-like cells. As shown in Figure 6A,B, the treatment of the HUVEC with VEGF (20 ng/mL) activated all three MAPKs including p38, ERK and SAPK/JNK, but DBHA markedly suppressed the VEGF-induced phosphorylation of p38 and JNK. The activation of ERK was not greatly affected by DBHA. These data suggest that the inhibition of the migration, proliferation and tube formation of the HUVEC is in part associated with the suppression of the VEGF-stimulated activation of the p38 and JNK signaling pathways. The activation of p38 was down-regulated dramatically by the treatment of DBHA (0–10 μM) in the mES/EB-derived endothelial-like cells. In addition, the activation of the PI3K/AKT/mTOR pathway contributes to the VEGF-mediated stimulation of proliferation and migration of endothelial cells [28]. Therefore, we also investigated the effect of DBHA on the VEGF-induced activation of the PI3K/AKT/mTOR signaling pathway in the HUVEC and the mES/EB-derived endothelial-like cells. DBHA effectively suppressed the activation of the PI3K and AKT kinases. In addition, the suppression of PI3K and Akt by DBHA subsequently led to a blockade of the activation of mTOR and its downstream effectors p70S6K and 4EBP1 (Figure 6C,D). To further confirm the association of DBHA with p38 and PI3K signaling pathway, the effects of p38 inhibitor SB203580 and PI3K inhibitor LY294002 were evaluated. As shown in Figure 6E,F, DBHA exhibits the similar suppression pattern with p38 and/or PI3K inhibitor in HUVEC and the mES derived endothelial-like cells. In addition, the co-treatment of p38 or PI3K inhibitor with DBHA also enhanced the suppression of the activation of p38 and PI3K signalings. These data suggest that the suppression of the endothelial cell biomarker PECAM by DBHA is in part mediated by the down-regulation of MAPKs and the PI3K/AKT/mTOR signaling in HUVEC and the mES/EB-derived endothelial-like cells. 

### 2.6. Global Discussion

Modern pharmaceutical discovery programs owe much to natural products. Indeed, pharmacologically active compounds from plants, microbes and marine animals represent an important pipeline for new investigational drugs [29]. 6″-debromohamacanthin A (DBHA) possesses notable antibacterial activity [19]. DBHA also exhibits moderate to significant cytotoxicity against five human cancer cell lines [15]. The present study demonstrated that DBHA exerted anti-angiogenic activity through the suppression of tube formation, cell migration and endothelial cell proliferation in HUVEC (Figure 2A). Further investigation revealed that DBHA functioned as an angiogenesis inhibitor via the suppression of the VEGFR2-mediated signaling pathway (Figure 6). Consistent with our *in vitro* data, DBHA also inhibited capillary sprouting in mES cell-derived EBs and in the *ex vivo* mouse aortic ring assay (Figure 4, Figure 5).

Angiogenesis is the formation of new blood vessels from the endothelium of existing vasculature, and the inhibition of angiogenesis is associated with a significant delay in tumor growth [30]. Angiogenesis also plays essential roles in tumor invasion and metastasis [31]. Thus, anti-angiogenic therapy is currently one of the most promising and efficient therapies against cancer [32]. Although the anticancer activity of DBHA was not demonstrated in the present study, based on the present results involving the anti-angiogenic activity of DBHA, DBHA might represent a potential chemotherapeutic strategy for inhibiting tumor growth. 

MAPK signaling is considered one of the critical molecular events for the growth, survival and migration of vascular endothelial cells in VEGF-induced angiogenesis. VEGF activates three MAPKs, namely ERK, JNK and p38 MAPK [33,34]. The ERK activation results in an increased proliferation of endothelial cells [34,35], whereas p38 MAPK activation triggers actin-based cell motility [33]. JNK plays a significant role in both the proliferation and migration responses [34]. Our data support the notion that three MAPKs mediate VEGF-induced tube formation, but the inhibitory effects of DBHA were more closely associated with the suppression of VEGF-induced p38 and JNK activation (Figure 6A). During angiogenesis, the PI3K and Akt kinases are activated in endothelial cells by a variety of stimuli [36], and they regulate multiple critical steps by phosphorylating different downstream substrates, such as mTOR [37]. The mTOR kinases, a central regulator of cell metabolism, growth, proliferation and survival [38,39], is activated during various cellular processes, such as tumor initiation, progression and angiogenesis [10]. Further investigation in the present study revealed that DBHA suppressed the phosphorylation of PDK1, AKT, mTOR, p70S6K and 4EBP1 in endothelial cells (Figure 6C) and EB-derived endothelial-like cells (Figure 6D). This suppression may be a possible mechanism of action for the anti-angiogenic or anti-proliferative activity of DBHA in endothelial cells. 

mES cell-derived EBs differentiate into endothelial cells and form vascular-like structures [40,41]. Therefore, the mES/EB-derived 3D model is recognized as an excellent system to examine anti-angiogenic activity [41,42,43,44]. Recently, we demonstrated the anti-angiogenic activity of honokiol, a natural angiogenesis inhibitor, with the mES cell-derived EB system [13]. In the present study, we found that DBHA inhibited neovascularization by suppressing microvessel sprouting in mES cell-derived EBs. The anti-angiogenic activity of DBHA was confirmed by an *in vitro* angiogenesis sprouting model system using the mES/EB model [44]. Previously, we have found that mES cell-derived EBs matured in a type-I collagen gel and formed capillary-like structures, as observed by morphological changes (3-D culture) and the expression of PECAM (2-D culture). In this angiogenesis sprouting model system, we found that DBHA blocked the formation of the primitive vascular network structures in mES cell-derived EBs (Figure 4C). In addition, these data correlate well with the inhibition of capillary sprouting of endothelial cells by DBHA in the mouse aortic ring model system (Figure 5). These results, which demonstrate the suppressive effects of DBHA in both *in vitro* and *ex vivo* angiogenesis assays, are consistent with the potential anti-angiogenic activities of DBHA.

## 3. Experimental Section

### 3.1. Reagent

6″-Debromohamacanthin A (DBHA), isolated from the marine sponge *Spongosorites* sp., was dissolved in 100% DMSO [25]. A 20 mmol/L stock solution of DBHA was prepared and stored as small aliquots at −20 °C until needed. We purchased 3-(4,5-Dimethylthiazol-2-yl)-2,5-diphenyltetrazolium bromide (MTT), dimethyl sulfoxide (DMSO), gelatin, and HRP-conjugated anti-mouse and anti-rabbit antibodies from Sigma-Aldrich (St. Louis, MO, USA). Recombinant human VEGF (VEGF_165_) was obtained from R&D Systems (Minneapolis, MN, USA). Growth factor-reduced Matrigel and collagen type-I were purchased from BD Biosciences (San Jose, CA, USA). The phospho-specific antibodies anti-p38 (Thr^180^/Tyr^182^), anti-SAPK/JNK (Thr^183^/Tyr^185^**)**, anti-PI3K, anti-PDK1, anti-AKT (Ser^473^), anti-mTOR (Ser^2448^), anti-p70S6K (Thr^389^), anti-4EBP1 (Thr^37^/Thr^46^), SB203580 and LY294002 were purchased from Cell Signaling Technology (Danvers, MA, USA). The HRP-conjugated β-actin, phospho-ERK (Thr^202^/Tyr^204^) and PECAM antibodies were purchased from Santa Cruz Biotechnology (Santa Cruz, CA, USA). Alexa Fluor 594-labeled chicken anti-rat IgG was purchased from Molecular Probes (Invitrogen, Carlsbad, CA, USA).

### 3.2. Endothelial Cell Culture

Human umbilical vascular endothelial cells (HUVEC) were obtained from ATCC (Rockville, MD, USA) and cultured in endothelial growth medium-2 (EGM-2) (Lonza, Walkersville, MD, USA) supplemented with 10% FBS at 37 °C in a 5% CO_2_ atmosphere. HUVEC at passages three to five were used in the experiments. The commercially available vascular endothelial cell-specific supplement EGM^®^-2 MV Bullet Kit was used [45]. 

### 3.3. Culture and Differentiation of Mouse Embryonic Stem Cells

Mouse D_3_ ES cells (ATCC, Rockville, MD, USA) were co-cultured with mitomycin C-treated mouse embryonic fibroblast (MEF) cells in high glucose DMEM (Invitrogen, Carlsbad, CA, USA) containing 15% fetal bovine serum (Hyclone, Ogden, UT, USA), 1000 U/mL of LIF/ESGRO (Chemicon, Temecula, CA, USA), and basic ES cell medium components [50 U/mL of penicillin and 50 μg/mL streptomycin (Invitrogen, Carlsbad, CA, USA), 1% non-essential amino acids (Invitrogen, Carlsbad, CA, USA) and 0.1 mM β-mercaptoethanol (Invitrogen, Carlsbad, CA, USA)]. The hanging drop method (20 μL per drop, 1 × 10^5^ cells/mL) was used to induce differentiation, as described by Heuer and colleagues [46] with minor modifications. The EBs were formed by incubating the hanging drop cultures for three days. The EBs were then transferred into the gelatin-coated wells of chamber slides (Nunc, Denmark) or 60 mm dishes to allow for attachment. Endothelial cell differentiation was induced in 3-day-old EBs by switching culture conditions to medium containing 5% FBS and growth factor cocktail (EGM2-MV Bullet Kit; Lonza, Walkersville, MD, USA).The viable cells were observed directly with an inverted phase contrast light microscope (Olympus Optical Co., Ltd., Tokyo, Japan). 

### 3.4. Cell Viability Assay

Cell viability was assessed by an MTT assay. HUVEC (5 × 10^3^ cells/well) were seeded into a 96-well plate with EGM-2 medium supplemented with 10% FBS. After allowing, the culture medium was removed, and the cells were rinsed twice with phosphate buffered saline (PBS) and then incubated with serum-free medium for 12 h. Following serum starvation, the cells were cultured in fresh 2% FBS medium containing various concentrations of DBHA at 37 °C for 3 days in the presence or absence of VEGF (20 ng/mL). The cytotoxicity in the endothelial-differentiated mES cells was determined without mLIF as previously described [47]. Briefly, cells (2 × 10^3^/well) were seeded into a 96-well plate and grown in the presence of test compounds. The cells were incubated for 24 h with DBHA (0–40 μM) 11 days after the induction of differentiation. After the incubation, an MTT solution was added, and the plate was incubated for an additional 4 h. The resulting formazan deposit was dissolved with DMSO, and the absorbance was detected at 570 nm with a VersaMax ELISA microplate reader (Molecular Devices, Sunnyvale, CA, USA). Cell viability was expressed as the percentage of surviving cells relative to the untreated cultures, and then the concentration required to inhibit cell growth by 50% (IC_50_) was calculated. The IC_50_ values were calculated using a non-linear regression analysis of percent-growth *versus* concentration. 

### 3.5. Scratch-Wound Migration Assay

The HUVEC were allowed to grow to full confluence in 6-well plates pre-coated with 0.1% gelatin and then incubated with 10 mg/mL mitomycin C (Sigma, St. Louis, MO, USA) at 37 °C in a 5% CO_2_ atmosphere for 2 h to inactivate the HUVEC. Monolayers HUVEC were wounded by scratching with a 0.2-mL pipette tip. Fresh medium containing various concentrations of DBHA was added. Images were taken with an inverted phase contrast light microscope (Olympus Optical Co., Ltd., Tokyo, Japan) after 24 h incubation. The migrated cells were then counted from three randomly selected fields under an optical microscope at 200× magnification. The migrated cells were quantified by manual counting (DMC advanced program), and the inhibition was calculated as a percentage relative to control. 

### 3.6. Transwell Migration Assay

The chemotactic motility of the HUVEC was determined using a transwell migration assay (Corning incorporated) with an 8-μm pore size as described elsewhere [48]. Briefly, the inserts of the transwell plate were coated with 0.2% gelatin for 30 min. After the transwells were washed three times with PBS, fresh EBM supplemented with 20 ng/mL VEGF was placed in the lower chamber and the HUVEC (4 × 10^4^ cells/well) were seeded in the top chamber. Then, the cells were treated with DBHA for 8 h at 37 °C in a 5% CO_2_ atmosphere. After the incubation, the non-migrated cells on the top surface of the membrane were gently scraped away with a cotton swab. The membrane containing the migrated cells was fixed with 4% paraformaldehyde for 10 min and stained with hematoxylin. Images were recorded using an OLYMPUS inverted microscope, and the migrated cells were quantified by manual counting. The percentage of migrated cells inhibited by DBHA was normalized to the untreated control cell migration.

### 3.7. Tube Formation Assay with HUVEC Cells on Matrigel

Matrigel (70 μL/well) was added to a 96-well plate and polymerized for 30 min at 37 °C. The HUVEC (3 × 10^4^ cells) were seeded onto each well of the Matrigel-coated 96-well plate and then incubated in 2% FBS-EBM-2 with various concentrations of DBHA in the presence of VEGF (20 ng/mL). After 8 h of incubation, the formation of endothelial cell tubular structure was visualized under an inverted microscope and photographed at 40× magnification. Furthermore, tube formation was quantified by calculating the tube length and was expressed as a percentage by normalization with untreated control cells. 

### 3.8. Western Blot Analysis

The HUVEC and mES/EB-derived endothelial-like cells were treated with various concentrations of DBHA for 24 h. The harvested cells were lysed in protein extraction solution (Intron Biotechnology, Inc., Seongnam, Korea) containing protease inhibitors and phosphatase inhibitors for 10 min at 4 °C. The protein concentration was measured using the Bradford assay. Equal amounts (40 μg) of protein samples were subjected to 6%–15% SDS-PAGE. The separated proteins were transferred to PVDF membranes (Millipore, Bedford, MA), which were then incubated with primary antibodies diluted in 5% BSA in TBST (1:200–1:2000) overnight at 4 °C. The membranes were then washed three times with TBST and incubated with the corresponding secondary antibodies. Protein bands were detected with an enhanced chemiluminescence detection kit (Intron Biotechnology, Inc., Seongnam, Korea) and a LAS-1000 Imager (Fuji Film Corp., Tokyo, Japan). 

### 3.9. Immunocytochemistry

After mES/EB-derived endothelial-like cells incubated with DBHA for 24 h, the cells were fixed with 4% paraformaldehyde and incubated overnight at 4 °C. The cells were blocked with blocking solution containing 1% BSA in PBS for 30 min and then incubated with rat anti-mouse PECAM (1:100) (Santa Cruz Biotechnology, Santa Cruz, CA, USA) overnight at 4 °C. After washing, the cells were then incubated with Alexa Fluor 594-labeled chicken anti-rat IgG (1:1000) (Invitrogen, Carlsbad, CA, USA). After staining, the cover slips were mounted with medium containing 4′,6-Diamidino-2-Phenylindole (DAPI) (Vector Laboratories, Burlingame, CA, USA). All of the fluorescence images were observed under a Zeiss ApoTome microscope (Carl Zeiss, Jena, Germany).

### 3.10. 3-Dimensional Collagen Type-I Sprouting Angiogenesis Model

The 3-dimensional tube formation and sprouting angiogenesis model were performed in type-I collagen. Briefly, the EBs derived from mES cells were cultured in suspension containing EGM-2 medium for 7 days. The EBs were then plated in a type-I collagen solution and incubated in EGM-2 medium at 37 °C. The effects of DBHA on the vascular sprout were determined after incubation with DBHA for 4 days. Vascular sprout morphology was recorded with an OLYMPUS inverted microscope, and the migrated cells were quantified by manual counting. The percentage of migrated cells inhibited by DBHA was normalized to the untreated control cell migration.

### 3.11. Aortic Ring Assay

The mouse aortic ring assay was performed as previously described [49]. Forty-eight-well plates were covered with 150 μL of Matrigel and then incubated at 37 °C and 5% CO_2_ for 30 min. The aortas isolated from mice (Central Laboratory Animal Inc., Seoul, Korea) were cleaned of periadventitial fat and connective tissues and cut into 1–1.5 mm long rings. After rinsing with PBS, the aortas were placed in the Matrigel-covered wells and covered with an additional 200 μL of Matrigel. The artery rings were cultured in 1 mL of EGM without serum for 24 h, and then the medium was replaced with 1 mL of EGM containing supplements with vehicle or DBHA (0.1, 1 or 10 μM). The medium was replaced every 2 days with medium that had the same composition as described above. After 7 days, the microvessel growth was measured by taking photographs with the OLYMPUS inverted microscope (40× objectives). The length of the capillary was estimated using a phase-contrast microscope by measuring the distance from the cut end of the aortic segment to the approximate middle point of the capillary. The length of the capillary was measured using Adobe Photoshop software (DMC advanced program). Each value represents the average of 3–4 culture samples. 

### 3.12. Statistical Analysis

The results are expressed as the mean ± SD. Statistical significance was determined using a one-way analysis of variance (ANOVA) and Student’s *t*-test for paired data. A *P*-value of <0.05 was considered statistically significant. The calculations were performed using SPSS for Windows Version 10.0 (SPSS, Chicago, IL, USA).

## 4. Conclusions

In conclusion, we systematically demonstrated that DBHA could effectively inhibit endothelial cell proliferation, migration and tube formation *in vitro*. In addition, DBHA potently inhibited microvessel sprouting in mES cell-derived EBs *in vitro* and in the mouse aortic ring model *ex vivo*. Herein, we demonstrate for the first time that DBHA inhibits angiogenesis in endothelial cells and mES cell-derived endothelial-like cells by targeting the VEGFR2-mediated PI3K/AKT/mTOR signaling pathway. 

## Supplementary Files

Supplementary File 1 Supplementary Information (PDF, 235 KB)
