# Supplementary material for: 6″-Debromohamacanthin A, a Bis (Indole) Alkaloid, Inhibits Angiogenesis by Targeting the VEGFR2-Mediated PI3K/AKT/mTOR Signaling Pathways
Source: Mar Drugs. 2013 Apr 2;11(4):1087–103. doi: 10.3390/md11041087 (PMC3705390; doi:10.3390/md11041087)

Supplementary Information

Figure S1. The growth inhibitory effects of DBHA in human cancer cells. Cells were treated with various concentrations of DBHA for 72 h. Cell viability was determined by the SRB and MTT assays. The percentages of cell survival were calculated by comparison with the vehicle-treated control cells. Data are expressed as the mean ± SD. The IC_{50} values were calculated by non-linear regression analysis. SNU601: Stomach cancer cells, K562: Leukemia, MDA-MB-231: Breast cancer cells, SK-HEP-1: Liver cancer cells, HCT116: Colon cancer cells, A549: Lung cancer cells, A549-Pa: Paclitaxel-resistant lung cancer cells.

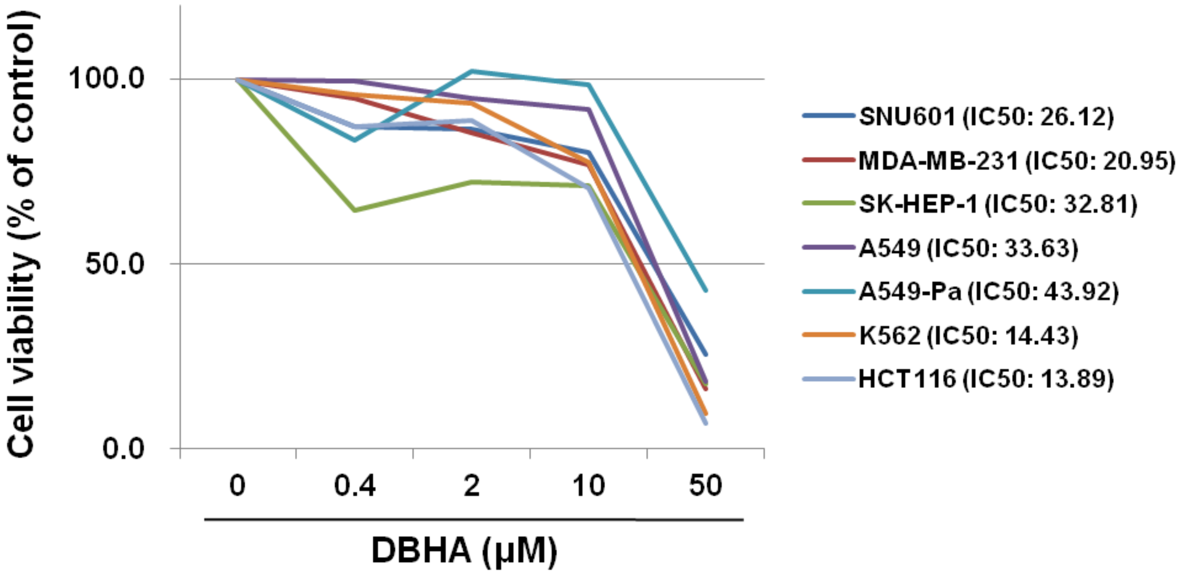

Figure S2. Sequential morphological and endothelial marker expression of ES-D_3 cells during the differentiation process. (**A**) ES-D_3 cells on CF1 feeder cells (a), Embryoid body (EB) on Day 3 after hanging drop culture (b), morphology on Day 4 (c) and Day 11 (d) of differentiated EBs after attachment to a gelatin-coated culture plate. Magnification: A 40×. (**B**) mES cells cultured for 4 days in 10^3 units/mL recombinant mouse LIF on a feeder layer of mouse embryonic fibroblasts (MEF, feeder cells) were fixed and tested for alkaline phosphatase activity with a colorimetric assay (a), immunofluorescent staining of mES cells with anti-SSEA-1 antibody (b). Magnification: B-a 200×, B-b 100×. (**C**) The expression of Oct4, a stemness marker, in endothelial precursor cells derived from mouse embryonic stem cells. The expression of Oct4 gradually decreased during endothelial differentiation. Analysis of the expression of endothelial cell-specific markers by RT-PCR and immunofluorescence in mES-derived embryoid bodies. (**D**) Day 0 corresponds to mRNA from mES cells cultured in the presence of LIF. Days 4 through 11 correspond to the numbers of days in culture after LIF deprivation. GAPDH served as an internal control. (**E**) mES/EB cells were differentiated into endothelial cells, fixed on days 4 (a) and day 11 (b) and stained with PECAM antibody. Magnification: E 100×.

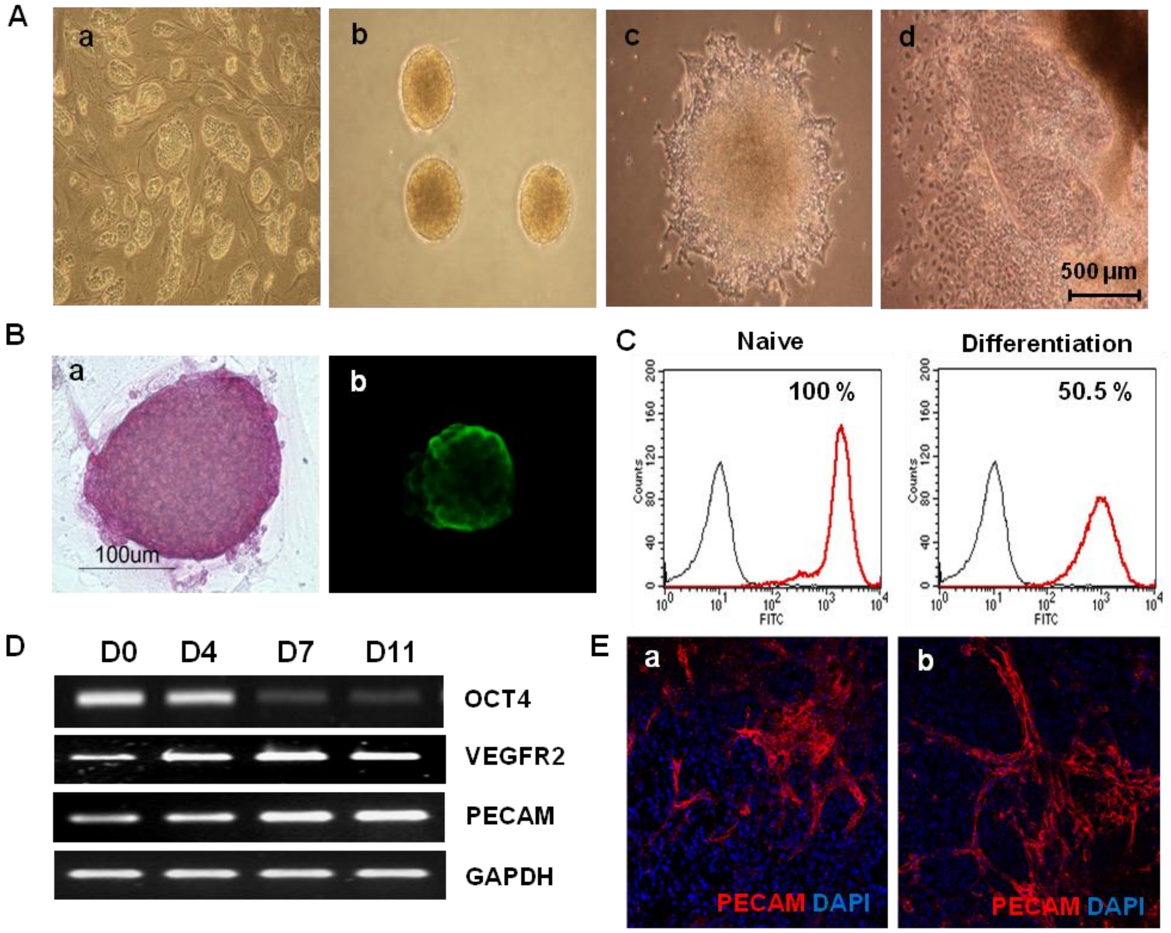

Supplement: Supplementary File 1 — Supplementary Information (PDF, 235 KB) [file marinedrugs-11-01087-s001.pdf]
